# Importance of *Aspergillus*-Specific Antibody Screening for Diagnosis of Chronic Pulmonary Aspergillosis after Tuberculosis Treatment: A Prospective Follow-Up Study in Ghana

**DOI:** 10.3390/jof9010026

**Published:** 2022-12-23

**Authors:** Bright K. Ocansey, Benjamin Otoo, Hafisatu Gbadamosi, Jane S. Afriyie-Mensah, Japheth A. Opintan, Chris Kosmidis, David W. Denning

**Affiliations:** 1 Division of Evolution, Infection and Genomics, Faculty of Biology, Medicine and Health, University of Manchester, Manchester Academic Health Science Centre, Manchester M13 9NT, UK; 2 Department of Bacteriology, University of Wisconsin-Madison, Madison, WI 53706, USA; 3 Radiology Department, Korle-Bu Teaching Hospital, Accra GA-221-1570, Ghana; 4 Chest Diseases Unit, Department of Medicine, Korle-Bu Teaching Hospital, Accra GA-221-1570, Ghana; 5 Department of Medicine and Therapeutics, University of Ghana Medical School, Accra GA-221-1570, Ghana; 6 Department of Medical Microbiology, University of Ghana Medical School, Accra GA-270-4330, Ghana; 7 National Aspergillosis Centre, Wythenshawe Hospital, Manchester University NHS Foundation Trust, Manchester M23 9LT, UK

**Keywords:** *Aspergillus* antibody, chronic pulmonary aspergillosis, Ghana, tuberculosis

## Abstract

Chronic pulmonary aspergillosis (CPA) often occurs in patients that have been previously treated for pulmonary tuberculosis (PTB). A limited number of studies have looked at the development of CPA at different times following the completion of a PTB treatment course. This prospective longitudinal study aimed to determine the incidence of CPA at two timepoints, at the end of the PTB treatment (T_1_) and six months post-treatment (T_2_). Patients with confirmed PTB from a previous study who were placed on anti-TB medication were followed up and screened for CPA at T_1_ and T_2_ by assessing their symptoms, evaluating their quality of life, and screening them for *Aspergillus* infection by performing antibody testing and cultures. CPA was defined by the Global Action for Fungal Infections (GAFFI) diagnostic algorithm. Forty-one patients were enrolled, of whom thirty-three patients (80%) and twenty-eight patients (68%) were resurveyed at T_1_ and T_2,_ respectively. The rate of new CPA was 3.3% (1/33) and 7.4% (2/27) at T_1_ and T_2_, respectively, with an overall incidence of 10.7% (3/28) among the patients at both T_1_ and T_2_. A positive *Aspergillus*-specific antibody test was an indicator for CPA in all three patients. *Aspergillus*-specific antibody screening during and after the end of an anti-TB treatment regimen may be important for early detection of CPA in high-PTB-burden settings.

## 1. Introduction

Pulmonary tuberculosis (PTB) remains a major global health problem, with a high burden in low- and middle-income countries (LMICs). In 2020, there were 4.8 million people diagnosed with PTB globally, and 59% of these cases had been bacteriologically confirmed [1]. About 85% of the people diagnosed with PTB are generally successfully treated with a 6 month drug regimen [1]. Unfortunately, patients with PTB after a successful treatment become more exposed to secondary respiratory infections that are uncommon in patients without prior PTB [2]. These infections can be chronic, and they are associated with high morbidity and mortality rates. Chronic pulmonary aspergillosis (CPA) is one of the common infections, and the high-risk group are patients with PTB with overt lung cavities [3]. The prevalence of CPA as a sequel of PTB worldwide was estimated at 1.2 million in 2011 [4]. Several cross-sectional studies have been conducted on CPA as a secondary infection of PTB in different countries, and they have reported varying rates [5,6,7,8,9]. However, none of these studies have solely focused on bacteriologically confirmed PTB, and so, they may have included individuals without prior PTB, and this may not be a true definition of the post-PTB complications. In recent times, there has been an interest in evaluating the timing of the incidence of CPA from diagnosis, during treatment and to post-treatment of PTB [3,10,11]. Such data could help guide screening strategies for early CPA diagnosis.

In addition, CPA may rarely co-exist with PTB as a primary CPA-PTB co-infection [12,13,14]. Genetic and environmental factors may also influence the development of CPA at different times among individuals [10,15]. Presently, CPA has been identified in PTB patients from the beginning of the PTB treatment and close to 40 years after completing the treatment [3,10,16]. The current prospective longitudinal study was conducted to evaluate the incidence of CPA at two timepoints following the completion of a PTB treatment regimen in a cohort of new bacteriologically confirmed PTB patients using *Aspergillus*-specific antibody testing as a screening tool.

## 2. Materials and Methods

Adult (≥18 years) patients with MTB detected in their sputum in a previous study [17] and were subsequently placed on an anti-TB treatment were enrolled for this study. The patients were receiving care for suspected PTB at the Chest Clinic of the Korle-Bu Teaching Hospital, Accra, Ghana, or they were referred from various health facilities across the country to the Chest Clinic TB laboratory for GeneXpert MTB and rifampicin (RIF) resistance testing (Xpert^®^ MTB/RIF, Cepheid, California, CA, USA). Patients with a previous history of PTB were excluded.

The patients’ demographics and baseline CPA screening findings including laboratory and chest radiograph reports were extracted from the primary research data. The patients were then followed-up and further screened for CPA at two timepoints; one was within one month after completing treatment (T_1_, 6–7 months from diagnosis) and the other was 6–7 months post-treatment (T_2_, 12–13 months from diagnosis). The CPA screening involved an assessment of the symptoms, *Aspergillus*-specific antibody testing, sputum *Aspergillus* culture, chest radiograph and/or a computed tomography (CT) scan.

Serum samples were obtained from all the patients for *Aspergillus*-specific antibody testing with the LDBio *Aspergillus* IgG & IgM LFA (LDBio Diagnostics, Lyon, France) following the manufacturer’s instructions. A sputum *Aspergillus* culture was performed for all of the patients using a modified version of the high-volume culture method by inoculating an aliquot (1–2 mL) of undiluted sputum on Sabouraud dextrose agar, and then, it was incubated at 37 °C for up to 8 days [17]. A chest CT scan was performed for the patients with positive *Aspergillus* serology or cavitation on baseline chest radiograph with new or persistent respiratory symptoms. A consultant radiologist (HG) who was blinded to the clinical and laboratory data evaluated all imaging investigations. Xpert MTB/RIF and/or acid-fast bacilli (AFB) smear results were retrieved from the laboratory records. Additionally, the quality of life (QoL) of the patients were evaluated at both timepoints using the St. George’s Respiratory Questionnaire (SGRQ), which scores patients from 1 (excellent health) to 100 (very ill) [18,19]. CPA was defined using the Global Action for Fungal Infections (GAFFI) diagnostic criteria (2018) [20].

The data were analyzed with SPSS version 25 (IBM, New York, NY, USA) at a 5% significance level using Chi Square test. The summary statistics are presented using frequencies and percentages for the categorical variables. Fisher’s exact tests were employed to compare the proportions of the various characteristics of the patients recruited at both, timepoints.

## 3. Results

**Figure 1:**
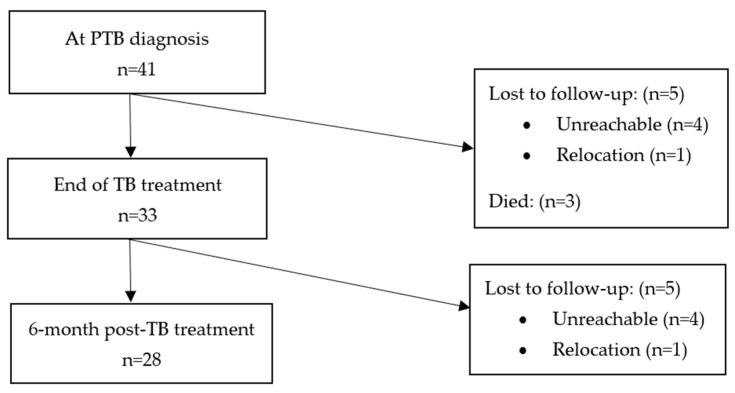
Overview of 41 patients enrolled.

There were 47 Xpert MTB positive cases, of whom, 41 were diagnosed as new cases. Of the 41 new PTB patients, 33 (80%) and 28 (68%) were resurveyed at T_1_ and T_2,_ respectively. In all, three of the thirteen patients who were not resurveyed at T_1_ and T_2_ died, and the remainder were lost during follow-up for varying reasons. Details of enrolled patients at both timepoints including those lost during follow-up are shown in Figure 1.

There were more male patients (78%, *n* = 32), and the mean age was 40.2 years (range, 18–75 years) (Table 1). Of the 41 positive Xpert MTB cases, majority of them (78%, *n* = 32) had a high or medium MTB load or concentration. No RIF resistance was recorded, but there were three cases of RIF indeterminate results (and so resistance could not be established). At baseline, *Aspergillus*-specific antibody was negative in all the patients. Cavitation was visible on chest radiograph in 18 patients at baseline. There were 10 patients whose sputum grew *Aspergillus* spp. All the 41 patients were placed on a standard first-line anti-TB regimen, comprising rifampicin, isoniazid, pyrazinamide, and ethambutol. The baseline SGRQ score was higher than 50 in 32% (13/41) of patients, with an average of 43.1 (Table 1).

**Figure 2:**
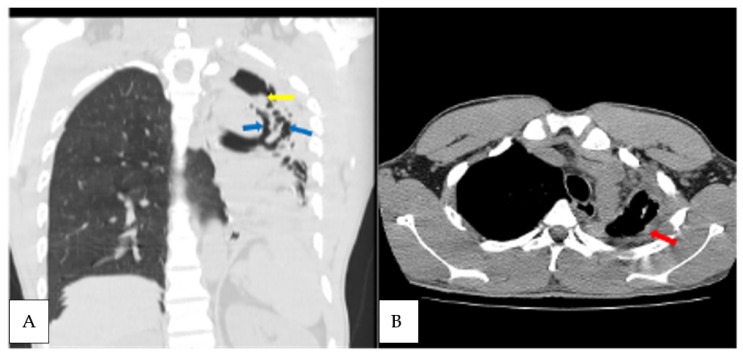
Axial non contrast CT scan, coronal reformatted lung, and axial mediastinal windows of the chest of the patient diagnosed with CPA at T_1_ (**A**). Extensive left lung traction bronchiectasis (blue arrows) with ipsilateral lung volume loss, left apical lung cavity with intracavitary material (yellow arrow); (**B**) left apical lung pericavitary pleural thickening (red arrow).

Of the 33 patients resurveyed at T_1_, 90.9% of them (30/33) had completed TB treatment and achieved microbiological cure (AFB smear negative), and three patients were declared to have failed the treatment due to persisting symptoms and a positive AFB smear. The *Aspergillus*-specific antibody was positive in one patient who was presumed to have failed the treatment due to persisting symptoms, but his AFB smear was negative, and he was placed on a second-line treatment. This patient met the criteria for CPA with suggestive image findings on a CT scan (Figure 2) and sputum growing *A. fumigatus* and *A. niger* (Table 2). The SGRQ score improved significantly at T_1_ for patients without CPA or treatment failure, with an average score of 51.4 falling to 3.8, signifying PTB treatment success. The SGRQ for the CPA patient decreased from 45 at baseline to 29 at T1. The three patients who failed TB treatment had a SGRQ reduction from an average of 51 to 26.3, respectively.

**Figure 3:**
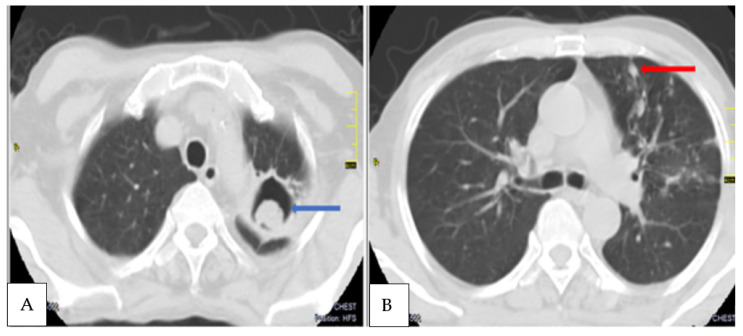
Axial contrast CT scans, axial lung window of chest of another CPA patient diagnosed at T_2_ showing (**A**) a soft tissue mass within a left apical lung cavity (blue arrow) with a characteristic crescent of air around it, the ‘Monod sign’, indicative of an aspergilloma. (**B**). Nodular opacities (red arrow) and non pericavitary fibrotic changes in the left upper lobe.

**Figure 4:**
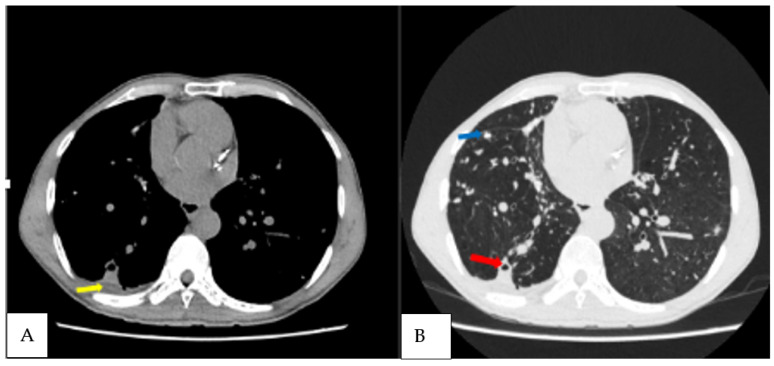
Axial non contrast CT scan, axial mediastinal and lung windows of the chest of the third CPA patient diagnosed at T_3_ patient, demonstrating (**A**) right lung nodules (blue arrow); (**B**) small right lower lobe cavity with pericavitary infiltration (red arrow) as well as a small pleural effusion (yellow arrow).

The 28 patients rescreened at T_2,_ included the previous CPA patient. Twenty-seven patients had achieved microbiological cure, while one was confirmed as having had a PTB relapse. Of the 27 patients with a previous negative *Aspergillus* antibody test, 7.4% of them (2/27) seroconverted. These two new patients also met the criteria for CPA, with one of them having a fungal ball (Figure 3 and Figure 4), and both had *A. fumigatus* growing from sputum. One of the CPA patients was HIV positive. Again, the two new patients who developed CPA at T_2_ were symptomatically worse with increasing SGRQ scores from 14.5 at T_1_ to 36 at T_2._ Generally, among the patients resurveyed at both time points, the average SGRQ scores for those without CPA had improved significantly compared to those with CPA. The other reasons for a higher SGRQ score were anti-TB treatment failure and PTB relapse.

The overall incidence of CPA among the resurveyed patients over 12 months from the time of PTB diagnosis was 10.7% (3/28). The incidence of CPA at end of the PTB treatment (T_1_) was 3% (1/33), which later increased to 7.4% (2/27) at 6 months post-treatment (T_2_). This represents 42.9% (3/7) of the patients with new or persisting symptoms suggestive of PTB after either 6 months or 12 months post-initial PTB diagnosis. Haemoptysis was only noted in patients who had CPA when they were resurveyed at T_1_ and T_2_. The common CT scan findings among the three CPA patients were cavitation, pleural thickening, pericavitary fibrosis and bronchiectasis (Table 2). Two of the three CPA patients had cavitation based on a baseline chest radiograph, representing 11.1% (2/18) of all of those with cavitation.

## 4. Discussion

We prospectively followed up new bacteriologically confirmed PTB patients from a previous study [17] to evaluate the incidence of CPA from the time of PTB diagnosis to the time after completing the TB treatment, and finally, six months after the treatment regimen. A CT scan was performed in all the CPA patients in our study. This makes the diagnosis more robust because a CT scan is very important for confirming a CPA diagnosis with a maximum score point in the recently published CPA EQUAL scores [21]. This study adds to previous studies that have indicated the need to regularly screen for CPA post-PTB treatment [3,5,10]. It also contributes to the emerging evidence that CPA may develop during or early after completion of anti-TB medications, as previously reported [10]. The current study also shows the increasing incidence rate of CPA among post-PTB patients when they are resurveyed over time, which is similar to previous findings [10].

Interestingly, at the baseline, no patient met the criteria for CPA, that is, we found no PTB-CPA coinfection at the time of the confirmed PTB diagnosis. However, it possible for CPA to develop two months after initiating anti-TB treatment, as reported by two recently published studies in Indonesia [22] and in Uganda [6]. First, Setianingrum et al. [10] in Indonesia, reported a CPA incidence of 7.9% among PTB patients who were within 2 months into an anti-TB treatment. It is important to note, however, that their cohort included at least 42% new non-bacteriologically confirmed PTB cases, and some of them probably had CPA alongside other respiratory disorders, including asthma and chronic pulmonary obstructive disease (COPD) as an underlying condition. Similarly, another study in Uganda reported ~20% CPA incidence among PTB patients with persisting symptoms after 2 months of TB treatment [6]. In a recently published study, a possible PTB-CPA coinfection was reported, but there are still doubts with regard to the existence of a true PTB infection [17]. As more studies are being conducted on CPA in patients presenting with PTB, the phenomenon of co-infection may be further explored.

In the current study, the T_1_ CPA patient resurveyed after six months, continued to have features of CPA including a positive *Aspergillus*-specific antibody test and *Aspergillus* culture which were accompanied by a worsening SGRQ score. Similar observations have been made in other studies [3,10]. However, it has been reported that some of these patients may no longer have the features of CPA when they are resurveyed without any antifungal treatment, surgical procedure or radiotherapy [10]. The data available on this phenomenon are scanty, and notwithstanding, it is widely accepted that some cases of CPA may be self-resolving or remain static for long periods [13].

*Aspergillus* specific-antibody testing was critical in identifying potential cases of CPA and distinguishing them from treatment failures or PTB recurrent cases with CT scan investigations. It is worthy to note that, all the PTB patients who were eventually diagnosed with CPA had a positive *Aspergillus*-specific antibody test, and was helpful in indicating, the need for advanced imaging seeking radiological features suggestive of CPA. *Aspergillus*-specific antibody can be detected by several methods including, precipitins, counter immuno-electrophoresis, immunodiffusion, complement fixation, enzyme immunoassay or immunochromatography. All of these tests have limitations including, false positives and false negatives [22,23]. In fact, evaluation studies and the clinical use evaluations of the LDBio *Aspergillus*-specific IgG and IgM assay employed in our study have reported varying sensitivities and specificities pooled at 90% and 91%, respectively, among different populations [6,8,24,25,26,27,28]. Although, an elevated *Aspergillus*-specific IgG level is superior compared to other immunoglobulins in the diagnosis of CPA, it is possible for some CPA patients to have normal levels of *Aspergillus*-specific IgG but raised levels of *Aspergillus*-specific IgM, which may be positive in about half of the CPA patients [22]. Thus, an *Aspergillus*-specific IgG and IgM assay may offer additional sensitivity. Like *Aspergillus*-specific IgM, *Aspergillus*-specific IgA and IgE can also be positive when the symptoms and imaging features suggestive of CPA are observed and the *Aspergillus*-specific IgG levels are normal. *Aspergillus*-specific IgE may be linked to allergic aspergillosis as the underlying condition for CPA, but it also may be independently elevated.

The performances of some *Aspergillus*-specific antibody assays are negatively affected by the relatively common minor or subtle immunodeficiencies found in CPA patients. However, the LDBio *Aspergillus*-specific IgG and IgM assay has been previously reported to be minimally affected by immunodeficiency, and thus, it may perform acceptably well in both HIV seronegative and seropositive patients [29]. Notwithstanding, CPA is rarely associated with HIV, and it is more common in patients without apparent or with subtle immunodeficiency. Our study suggests the LDBio *Aspergillus*-specific IgG and IgM can be used with the GAFFI diagnostic algorithm, in accord with a previous report from Uganda [27]. However, the algorithm relies mainly on chest radiographs, which would have missed one CPA patient in this series who had CPA-suggestive image findings demonstrated only on CT scan images. Clinicians thus need to consider *Aspergillus*-specific antibody testing in successfully treated PTB patients who return with new or persistent respiratory symptoms without radiological progression on chest radiographs. Most of these cases are usually presumed to have anti-TB treatment failure, PTB relapse or reinfection, but studies have shown that over 50% actually have CPA [9,17,30]. The current finding corroborates a recent study in Ghana, where CPA was present in 50% of the patients with presumed recurrent PTB [17]. Although the *Aspergillus* culture was positive in eight more patients at T_1_, none of them had symptoms or a previous chest radiograph suggestive of CPA, and so culture alone should not be used to diagnose CPA in the absence of characteristic radiological findings.

The major limitation of our study was that our sample size was small, and thus, it may not be sufficiently representative. A validation in a larger population will carry more statistical weight. Additionally, some of the patients lost during follow-up may have developed CPA, and thus, we may have underreported the frequency of this problem as in other studies with a significant proportion of study subjects who were lost during follow-up.

## 5. Conclusions

The present study indicates that CPA may develop during and after completing an anti-TB treatment regimen among new bacteriologically confirmed PTB patients. *Aspergillus*-specific antibody testing is instrumental in screening patients prior to performing CT scans to confirm the cases of CPA in resource-constrained settings, where advanced imaging is mostly unavailable or expensive to access. We recommend the validation of these findings in a larger cohort study. Additionally, subsequent studies may also consider investigations at 3 months or half of the duration into anti-TB treatment regimen. This will be important to contribute to efforts in identifying strategies for early detection of CPA cases, particularly in high-PTB-burden settings to minimize the number of inappropriate retreatments for PTB and late presentations with aspergilloma.

## Figures and Tables

**Table 1:** Patients’ characteristics at the three time points.

Features	Baseline (*n* = 41)	End of PTB Rx (*n* = 33)	6-Month Post-PTB Rx (*n* = 28)	*p*-Value
**Demographics**				
Male	32 (78%)	27 (81.8%)	23 (82.1%)	
Female	9 (22%)	6 (18.2%)	5 (17.9%)	<0.001
Age (mean/range)	40.2 (18–75)	41.2 (18–75)	39.7 (18–75)	0.962
**Symptoms**				
Haemoptysis	9 (22%)	2 (6.1%)	2 (7.1%)	<0.001
Chest pain	19 (46.3%)	4 (12.1%)	6 (21.4%)	0.253
Dyspnea	16 (39%)	1 (3%)	1 (3.6%)	0.071
Fatigue	33 (80.5%)	6 (18.2%)	2 (7.1%)	<0.001
Weight loss	36 (87.8%)	6 (18.2%)	3 (10.7%)	<0.001
**Laboratory**				
MTB load distribution				
Trace	2 (4.9%)	-	-	
Very low	3 (7.3%)	-	-	
Low	4 (9.8%)	-	-	
Medium	9 (22%)	-	1 (3.6%)	
High	23 (56.1%)	-	-	<0.001
+*Aspergillus* serology	0	1 (3%)	3 (10.7%)	<0.001
+*Aspergillus* culture	10 (24.4%)	9 (27.3%)	8 (28.6%)	<0.001
*Aspergillus* spp. distribution				
*A. fumigatus*	8 (16%)	6 (18.2%)	4 (14.3%)	
*A. flavus*	1 (2.4%)	0	1 (3.6%)	
*A. niger*	4 (9.7%)	5 (15.1%)	4 (14.3%)	
*A. terreus*	0	1 (3%)	0	<0.001
+HIV serology	12 (24%)	8 (23.5%)	6 (21.4%)	0.003
+AFB smear	-	3 (9.1%)	0	<0.001
**Imaging**				
Cavity	9 (22%)	6 18.2%)	4 (14.3%)	<0.001
Infiltration	14 (34.1%)	3 (9.1%)	2 (7.1%)	0.020
Fibrosis	25 (60.9%)	4 (12.1%)	6 (21.4%)	<0.001
Pleural thickening	20 (48.8%)	4 (12.1%)	4 (14.3%)	0.333
Bronchiectasis	19 (46.3%)	2 (6.1%)	3 (10.7%)	0.252
Fungal ball	0	0	1 (3.7%)	<0.001
**Quality of Life**				
SGRQ (mean ± SD)	43.1 ± 9.127	9.3 ± 6.848	13.1 ± 18.064	0.124

+—positive; Rx—Treatment; HIV—human immunodeficiency virus; SD—standard deviation.

**Table 2:** Demographics, symptoms, laboratory, imaging and QoL details of the three CPA patients.

Age	Sex	Symptoms	ASPG Ab	Culture	CT Scan at T_1_ /T_2_	SGRQ at Baseline, T_1,_ and T_2_
30	M	Cough, haemoptysis, chest pain, weight loss	Positive	*A. fumigatus*, *A. niger*	Cavities, intracavitary material, pericavitary infiltration, pleural thickening adjacent cavity, bronchiectasis, pleural effusion	45, 29, 83
49	M	Cough, dyspnoea, weight loss	Positive	*A. fumigatus*	Cavities, fungal ball, pleural thickening adjacent cavity, pericavitary fibrosis, bronchiectasis	46, 14, 23
50	M	Haemoptysis, cough, fatigue	Positive	*A. fumigatus*	Cavities, intracavitary material, pleural thickening adjacent cavity, pericavitary fibrosis, nodules, bronchiectasis	30, 15, 49

ASPG—*Aspergillus*; Ab—antibody; M—male.

## Data Availability

The research data associated with this paper are available from the corresponding author upon reasonable request.
